# Continuing professional development (CPD) training needs assessment for medical laboratory professionals in Ethiopia

**DOI:** 10.1186/s12960-023-00837-1

**Published:** 2023-06-20

**Authors:** Endale Hadgu Gebregzabher, Firehiwot Tesfaye, Waqtola Cheneke, Abebe Edao Negesso, Gizachew Kedida

**Affiliations:** 1 Department of Biochemistry, St. Paul’s Hospital Millennium Medical College, Addis Ababa, Ethiopia; 2 School of Medicine, Diredawa University, Diredawa, Ethiopia; 3 School of Medical Laboratory Science, Jimma University, Jimma, Ethiopia; 4 Department of Medical Laboratory Sciences, Addis Ababa University, Addis Ababa, Ethiopia; 5 Ethiopian Medical Laboratory Association (EMLA), Addis Ababa, Ethiopia

**Keywords:** CPD, Laboratory professionals, Ethiopia, Africa

## Abstract

**Background:**

Continuing professional development (CPD) is required for health workers in practice to update knowledge and skills regularly to match the changing complexity of healthcare needs. The objective of this study was to identify the training needs of Medical Laboratory professionals in Ethiopia.

**Methods:**

A total of 457 medical laboratory professionals from five regions and two city administrations were involved in the study. Data were collected from August 02 to 21, 2021 with structured self-administered online tool with five-point Likert scale. The tool had consent, demography, cross-cutting issues, and main activity area specific to medical laboratory.

**Results:**

Majority of the participants were male (80.1%). Participants from Amhara region 110 (24.1%) were the largest groups in the survey followed by Oromia 105 (23%) and Addis Ababa 101 (22.1%). The study participants comprised 54.7% with a bachelor’s degree, 31.3% with a diploma (associate degree), and 14% with a master’s degree. The participants had varying years of service, ranging from less than one year to over 10 years of experience. Most of the participants work as generalists (24.1%) followed by working in microbiology (17.5%) and parasitology (16%). The majority (96.9%) were working in a public sector or training institutions and the rest were employed in the private sector. Our study showed that the three most important topics selected for training in the cross-cutting health issues were health and emerging technology, computer skills and medico-legal issues. Topics under microbiology, clinical chemistry and molecular diagnostics were selected as the most preferred technical areas for training. Participants have also selected priority topics under research skill and pathophysiology. When the laboratory specific issues were regrouped based on areas of application as technical competence, research skill and pathophysiology, thirteen topics under technical competence, four topics under research skill and three topics under pathophysiology were picked as priority areas.

**Conclusion:**

In conclusion, our study identified that CPD programs should focus on topics that address technical competence in microbiology, clinical chemistry and molecular diagnostics. Additionally competencies in research skill and updating knowledge in pathophysiology should also receive due attention in designing trainings.

**Supplementary Information:**

The online version contains supplementary material available at 10.1186/s12960-023-00837-1.

## Background

Medical laboratory professionals are responsible for performing complex scientific procedures in areas like hematology, chemistry, virology, and microbiology, while conducting a variety of tests on blood, urine, body fluids, and tissues. They play a key role in today's healthcare environment by ensuring correct diagnosis and appropriate treatment of patients and also in improving overall practice of medicine. There is a universal consensus that many professional including those in the laboratory need to update and improve their knowledge and skills so that it does not become outdated or obsolete. Continuing professional development (CPD) in the medical field enables professionals to acquire knowledge, improve skills and performance to effectively function in an ever-changing healthcare environment [1–5]. CPD is the systematic maintenance, improvement and broadening of relevant knowledge and skills, and the development of these qualities is necessary for the professionals to successfully carry out their duties throughout their career. Continuous professional development is an integral requirement of both professional and health service providers in the form of lifelong education [1, 6].

CPD is a career development—long process that requires health professionals to enhance their knowledge, acquire new skills and build on existing ones [7, 8]. The main objective of CPD is to promote up-to-date and high-quality patient care by ensuring that health professionals have access to the necessary learning opportunities to maintain and improve their ability to practice [1, 8]. In sub-Saharan African countries, accessing CPD programs is a major challenge for laboratory and other health care personnel, partly due to their limited availability [5, 9, 10]. Many developed as well as developing countries are working hard to ensure that healthcare employees develop their knowledge and skill by creating policies and regulations for CPD. Because, a national CPD program has benefits for individual professionals, for regulatory bodies, for governments, and most importantly, for the public [11].

In Ethiopia, the Federal Ministry of Health acknowledged the gaps in CPD for all health professionals in its 2015–2020 Health Sector Transformation plan [12]. So far in Ethiopia, there were no standardization, regulation and accreditation of health workforce. Besides, CPD activities have never been linked to re-licensure of health professionals. In 2018 the FMOH finalized a CPD directive and guideline to fully implement starting from 2020 to 2021 [13]. Starting from 2018, CPD is mandatory to all health professionals wherever they work—in hospitals, clinics, health centers, training schools, universities, commercial companies, charities, orphanages, and schools, NGOs [13]. However, the implementation of the mandatory participation in CPD program has not been effected. Moreover, CPD activities in Ethiopia are fragmented as there are no standardization, regulation and accreditation mechanisms.

Therefore, the aim of conducting this study was to assess training needs for medical laboratory professional in Ethiopia and identify priority areas for training. Conducting a needs assessment may provide useful information for CPD providers, accreditors and course developers what priority areas should be selected for designing and delivering CPD courses to medical laboratory professionals and contribute in standardization and implementation of the CPD agendas set forward by the federal ministry of health. Needs assessments can also provide baseline information that can be used to monitor or evaluate CPD program.

## Methods

### Setting and participants

Ethiopia has ten regional states and two city administrations. This assessment was conducted over four selected regional states of Ethiopia (Oromia, Amhara, SNNPRS and Sidama) and two city administration (Addis Ababa and Dire dawa). In those selected regions, there are 31 teaching hospitals, and 16 regional health science colleges. Those selected regions and city administration were assumed to represent the largest workforce for the country and the information obtained from them can give clear picture of training of selected health professionals for designing of CPD courses. All medical laboratory technologists working at randomly selected health facility and regional science colleges were included in the study populations. This study was conducted on from August 2 to 21, 2021.

### Design and sample size

A cross-sectional survey of selected laboratory professionals was conducted. Sample size was calculated using single population proportion sample size estimation formula as previously described [Ref.]. For the calculation 95% CI, margin of error 5% and 50% population proportion was used and the sample size was 384. Proportional allocation to the size of health professional at regions was applied to divide the sample into the participating regions/city administrations. Finally, study facility was randomly selected to and included all health professionals working in the selected health facilities. We added 100 to compensate for non-response and the final sample size used was 484.

### Data collection

The data were collected online with structured questionnaire using the google forms. Data collection tool for this survey assessment was developed in context of medical laboratory profession and it has three sections: demographic, cross-cutting issues, and profession specific areas of CPD need. Each areas of tool was scored using the five-point Likert scale as 1 = need no support required or no gap of knowledge or skill: perform competently; 2 = need minimal support: little gap of knowledge/skill; 3 = need average level of support: some level of knowledge /skill gap; 4 = need moderate level of support: significant gap in knowledge/skill; and 5 = need high level of support: extensive gap in the knowledge or skill. Additionally, responders will be asked to rank areas for CPD course identification. At the same time, they were also asked to recommend potential areas for personal developments.

### Analysis

SPSS version 25 was used for analysis. Descriptive analysis (frequency, percentage, mean, range) of the 5-point Likert data was used to describe the CPD needs of the laboratory professionals. The top priority training needs under laboratory specific issues were identified using estimation of mean.

### Ethical considerations

The study obtained IRB ethical clearance from Yekatit 12 memorial hospital. Support letter was submitted and agreement was obtained from the responsible person at various levels of health systems. The data will only be used for guiding the intervention and determining the success of interventions.

## Results

### Characteristics of the study population

Four hundred and fifty-seven laboratory professionals responded online to the survey questionnaires which were conducted online using the Microsoft google form. Majority of the participants were male (80.1%). Participants from Amhara region 110 (24.1%) were the largest groups in the survey followed by Oromia 105(23%) and Addis Ababa 101(22.1%). The study participants comprised 54.7% with a Bachelor degree, 31.3% with a diploma (Associate degree), and 14% with a Master’s degree. The participants had varying years of service, ranging from less than one year to over 10 years of experience. Most of the participants work as generalists (24.1%) followed by working in hematology (21%) and microbiology (17.5%). The majority (96.9%) were working in a public sector laboratory or training/research institution and the rest were employed in the private sector (Table 1).

**Table 1:** Demographic characteristics of participants (*n* = 457)

	Frequency	Percentage
Gender		
Female	91	19.9
Male	366	80.1
Region		
Addis Ababa	101	22.1
Amhara	110	24.1
Dire dawa	14	3.1
Harari	30	6.6
Oromia	105	23
Sidama	24	5.3
SNNPR	73	16
Educational level		
Diploma/associate degree	143	31.3
Bachelor (BSc)	250	54.7
Masters (MSc)	64	14
Years of service		
< 1 year	4	0.9
1–5 years	105	23
6–10 years	174	38.1
> 10 years	73	16
Not specified	101	22.1
Current place of work		
Educational or research institution	59	12.9
General hospital	109	23.9
Health center	154	33.7
Primary hospital	44	9.6
Private health facility	14	3.1
Regional laboratory	15	3.3
Specialized hospital	62	13.6
Area of specialty within laboratory		
Blood bank	16	3.5
Clinical chemistry and molecular diagnostics	40	8.8
Generalist	110	24.1
Hematology	96	21
Immunology and serology	31	6.8
Microbiology	80	17.5
Parasitology	73	16
Urine and body fluid analysis	11	2.4

### CPD educational preferences of the laboratory personnel in cross-cutting health issues

The top three selected topics as most important for training in the cross-cutting health issues were health and emerging technology, most important (24.8%), computer skills (10.7%) and medico-legal issues (9.4%) (Table 2).

**Table 2:** Ranking of training needs in cross-cutting health issues, *n* = 457

Training needs	Ranking				
	Most important n (%)	Important *n* (%)	Moderate importance *n* (%)	Low importance *n* (%)	No importance *n* (%)
Cross-cutting health issues					
Communication skills in health and Customer care	22 (4.8)	17 (3.7)	65 (14.3)	135 (29.6)	217 (47.6)
Medico-legal issues	43 (9.4)	57 (12.5)	82 (18.0)	120 (26.3)	154 (33.8)
Infection Prevention and Control	21 (4.6)	25 (5.5)	91 (20.0)	129 (28.3)	190 (41.7)
Basic life saving skills	36 (7.9)	60 (13.2)	99 (21.7)	115 (25.2)	146 (32.0)
Gender in health service delivery	31 (6.8)	33 (7.2)	61 (13.4)	108 (23.7)	223 (48.9)
Gender based violence	34 (7.5)	35 (7.7)	64 (14.0)	119 (26.1)	204 (44.7)
Computer skills	49 (10.7)	52 (11.4)	113 (24.8)	133 (29.2)	109 (23.9)
Health and emerging technology	113 (24.8)	82 (18.0)	102 (22.4)	94 (20.6)	65 (14.3)

### CPD educational preferences in laboratory specific issues

The laboratory specific Likert data are grouped together into three areas: technical competence (9 themes), pathophysiology (1 theme) and research skill (1 theme) (Table 3). The tables summarize the responses of participants to each of the training topics selected for assessment under the pre-determined themes. To narrow down the possible number of topics which can potentially be picked to prepare training modules based on this study, we compared the mean scores of the topics under “most important” ranking in the Likert data. Based on their mean scale scores, our results in reference to the three laboratory specific issues ranked by participants as “most important” in rank order are presented as follows: (i) microbiology, most important (mean, range 16.7%, 4.6% to 23.5%); (ii) clinical chemistry and molecular diagnostics, most important (mean, range 15.0%, 9.4% to 20.4%); (iii) accreditation and quality management, most important (mean, range 7.7%, 5.0% to 15.8%); (iv) immunology and serology, most important (mean, range 7.4%, 3.7% to 20.4%); (v) laboratory management, leadership and coaching, most important (mean, range 7.2%, 5.0% to 11.4%); (vi) hematology, most important (mean, range 6.9%, 3.5% to 14.5%); (vii) parasitology, most important (mean, range 6.6%, 3.9% to 12.9%); (viii) immunohematology/blood bank, most important (mean, range 6.9%, 3.9% to 8.8%) (ix) urine and other body fluid analysis, most important (mean, range 6.8%, 3.9% to 15.1%) (Table 3).

**Table 3:** Ranking of training needs in technical competence according to pre-determined themes in laboratory specific issues, *n* = 457

Training needs	Ranking				
	Most important *n* (%)	Important *n* (%)	Moderate importance *n* (%)	Low importance *n* (%)	No Importance *n* (%)
Laboratory management, leadership and coaching					
Ethics and professionalism	25 (5.5)	33 (7.2)	76 (16.7)	135 (29.6)	187 (41.0)
Critical thinking and decision-making	23 (5.0)	30 (6.6)	96 (21.1)	146 (32.0)	161 (35.3)
Supervision and delegation	30 (6.6)	45 (9.9)	106 (23.2)	152 (33.3)	123 (27.0)
Data management, report writing and presentation skill	26 (5.7)	42 (9.2)	89 (19.5)	157 (34.4)	142 (31.1)
Basic cost accounting for clinical laboratory services	46 (10.1)	49 (10.7)	104 (22.8)	116 (25.4)	141 (30.9)
Management of resources and supplies	26 (5.7)	39 (8.6)	102 (22.4)	140 (30.7)	149 (32.7)
Medical equipment management	27( 5.9)	45 (9.9)	93 (20.4)	130 (28.5)	161 (35.3)
Work place Stress management and burn out prevention	37 (8.1)	62 (13.6)	106 (23.2)	135 (29.6)	116 (25.4)
Team building	28 (6.1)	34 (7.5)	89 (19.5)	142 (31.1)	163 (35.7)
Strategic planning	38 (8.3)	53 (11.6)	110 (24.1)	141 (30.9)	114 (25.0)
Rationale selection of testes	30 (6.6)	51 (11.2)	100 (21.9)	136 (29.8)	139 (30.5)
Training facilitation skill/ clinical laboratory teaching skills	52 (11.4)	67 (14.7)	98 (21.5)	140 (30.7)	99 (21.7)
Competency assessment	39 (8.6)	55 (12.1)	86 (18.9)	132 (28.9)	144 (31.6)
Accreditation and quality management					
Evaluation and selection of analytical methods and equipment	38 (8.3)	64 (14.0)	107 (23.5)	145 (31.8)	102 (22.4)
Definition, establishment, and use of reference ranges	36 (7.9)	50 (11.0)	92 (20.2)	128 (28.1)	150 (32.9)
Point of care testing	30 (6.6)	44 (9.6)	83 (18.2)	139 (30.5)	160 (35.1)
Method validation and verification	63 (13.8)	64 (14.0)	120 (26.3)	130 (28.5)	79 (17.3)
Specimen management	23 (5.0)	28 (6.1)	70 (15.4)	123 (27.0)	212 (46.5)
Accreditation standards (ENAO, ISO15189)	72 (15.8)	69 (15.1)	91 (20.0)	123 (27.0)	101 (22.1)
Use of internal quality control (IQC) and external quality assessment (EQA)	28 (6.1)	36 (7.9)	76 (16.7)	106 (23.2)	210 (46.1)
Quality system essentials for medical laboratory	28 (6.1)	42 (9.2)	72 (15.8)	136 (29.8)	178 (39.0)
Preparation of standard operational procedures	25 (5.5)	44 (9.6)	65 (14.3)	124 (27.2)	198 (43.4)
Laboratory policies	24 (5.3)	32 (7.0)	79 (17.3)	124 (27.2)	197 (43.2)
Techniques to identify and control sources of errors in laboratory procedures	23 (5.0)	35 (7.7)	104 (22.8)	142 (31.1)	152 (33.3)
Management of non-conformances in laboratory service	36 (7.9)	55 (12.1)	104 (22.8)	161 (35.3)	100 (21.9)
Preparation and storage of reagents	31 (6.8)	42 (9.2)	75 (16.4)	129 (28.3)	179 (39.3)
Hematology					
Perform and interpret Hemoglobin/hematocrit testing	19 (4.2)	13 (2.9)	31 (6.8)	73 (16.0)	319 (70.1)
Perform and interpret blood cell count	22 (4.8)	17 (3.7)	47 (10.3)	99 (21.8)	270 (59.3)
Perform and interpret WBC differential count	24 (5.3)	22 (4.8)	65 (14.3)	89 (19.5)	256 (56.1)
Perform and interpret RBC morphology assessment	24 (5.3)	46 (10.1)	96 (21.1)	128 (28.1)	162 (35.5)
Perform and interpret preparation and examination of thick and thin blood film	18 (3.9)	19 (4.2)	39 (8.6)	102 (22.4)	278 (61.0)
Perform blood collection	16 (3.5)	10 (2.2)	20 (4.4)	50 (11.0)	360 (78.9)
Perform and interpret Coagulation tests	66 (14.5)	68 (14.9)	93 (20.4)	102 (22.4)	127 (27.9)
Perform and interpret flow cytometry procedure	64 (14.0)	70 (15.4)	81 (17.8)	122 (26.8)	119 (26.1)
Immunohematology/blood bank					
Perform and interpret Blood typing and cross matching	18 (3.9)	17 (3.7)	34 (7.5)	84 (18.4)	303 (66.4)
Perform and interpret preparation of red cell suspension	34 (7.5)	39 (8.6)	89 (19.5)	94 (20.6)	200 (43.9)
Perform and interpret preparation and storage of blood products	40 (8.8)	30 (6.6)	78 (17.1)	104 (22.8)	204 (44.7)
Perform and interpret antibody screening and identification	34 (7.5)	34 (7.5)	76 (16.7)	99 (21.7)	213 (46.7)
Immunology and serology					
Perform and interpret specific and nonspecific treponemal tests	29 (6.4)	29 (6.4)	73 (16.0)	101 (22.1)	224 (49.1)
Perform and interpret ASO test	28 (6.1)	28 (6.1)	40 (8.8)	100 (21.9)	260 (57.0)
Perform and interpret Widal and Weil–Felix test	18 (3.9)	9 (2.0)	16 (3.5)	60 (13.2)	353 (77.4)
Perform and interpret rapid serological tests	17 (3.7)	10 (2.2)	20 (4.4)	70 (15.4)	339 (74.3)
Perform and interpret pregnancy testing	17 (3.7)	4 (0.9)	14 (3.1)	47 (10.3)	374 (82.0)
Perform and interpret ELISA testing	93 (20.4)	83 (18.2)	110 (24.1)	83 (18.2)	87 (19.1)
Parasitology					
Perform and interpret collection and preservation of stool	18 (3.9)	16 (3.5)	58 (12.7)	90 (19.7)	274 (60.1)
Perform and interpret direct/wet mount stool examination	18 (3.9)	13 (2.9)	18 (3.9)	64 (14.0)	343 (75.2)
Perform and interpret stool concentration technique	37 (8.1)	31 (6.8)	75 (16.4)	119 (26.1)	194 (42.5)
Perform and interpret Examination and Identification of parasites	20 (4.4)	13 (2.9)	33 (7.2)	79 (17.3)	311 (68.2)
Perform and interpret scotch-tape technique	59 (12.9)	57 (12.5)	102 (22.4)	127 (27.9)	111 (24.3)
Microbiology					
Perform and interpret gram staining	34 (7.5)	26 (5.7)	49 (10.7)	100 (21.9)	247 (54.2)
Perform and interpret acid fast staining (AFS)	21 (4.6)	13 (2.9)	25 (5.5)	65 (14.3)	332 (72.8)
Perform and interpret culture media preparation	82 (18.0)	101 (22.1)	109 (23.9)	81 (17.8)	83 (18.2)
Perform and interpret inoculation of microbiological media	87 (19.1)	86 (18.9)	119 (26.1)	78 (17.1)	86 (18.9)
Interpretation of bacterial and mycological colony characteristics	102 (22.4)	95 (20.8)	118 (25.9)	70 (15.4)	71 (15.6)
Identification of bacterial and mycological species from solid and liquid culture media	107 (23.5)	102 (22.4)	117 (25.7)	68 (14.9)	62 (13.6)
Perform and interpret antimicrobial susceptibility testing	100 (21.9)	93 (20.4)	108 (23.7)	71 (15.6)	84 (18.4)
Urine and other body fluid analysis					
Perform and interpret physical examination of urine	19 (4.2)	8 (1.8)	25 (5.5)	79 (17.3)	325 (71.3)
Perform and interpret chemical examination of urine	18 (3.9)	5 (1.1)	23 (5.0)	64 (14.0)	346 (75.9)
Perform and interpret microscopic examination of urine	18 (3.9)	16 (3.5)	38 (8.3)	81 (17.8)	303 (66.4)
Perform collection and examination of other body fluids	69 (15.1)	75 (16.4)	99 (21.7)	85 (18.6)	128 (28.1)
Clinical chemistry and molecular diagnostics					
Perform operation and maintenance of clinical chemistry machines	70 (15.4)	77 (16.9)	108 (23.7)	93 (20.4)	108 (23.7)
Perform and interpret renal function tests	46 (10.1)	45 (9.9)	79 (17.3)	89 (19.5)	197 (43.2)
Perform and interpret liver function test	47 (10.3)	43 (9.4)	80 (17.5)	89 (19.5)	197 (43.2)
Perform and interpret lipid profile testing	54 (11.8)	54 (11.8)	79 (17.3)	91 (20.0)	178 (39.0)
Perform and interpret hormonal assays	87 (19.1)	82 (18.0)	88 (19.3)	84 (18.5)	114 (25.1)
Perform and interpret electrolyte analysis	81 (17.8)	72 (15.8)	86 (18.9)	81 (17.8)	136 (29.8)
Perform and interpret DNA/RNA extraction and quantification	153 (33.6)	93 (20.4)	87 (19.1)	79 (17.3)	44 (9.6)
Perform and interpret nucleic acid amplification-based tests/PCR	134 (29.5)	80 (17.6)	83 (18.2)	78 (17.1)	80 (17.6)
*Ranking of training needs in pathophysiology according to pre-determined themes in Laboratory specific issues, n = 457*					
Pathophysiology					
Case studies in clinical microbiology	86 (18.9)	80 (17.5)	118 (25.9)	94 (20.6)	78 (17.1)
Case studies in clinical chemistry and molecular diagnostics	93 (20.4)	78 (17.1)	123 (27.0)	104 (22.8)	58 (12.7)
Case studies in hematology	77 (16.9)	63 (13.8)	94 (20.6)	126 (27.6)	96 (21.1)
Case studies in blood banking	68 (14.9)	70 (15.4)	105 (23.0)	109 (23.9)	104 (22.8)
Case studies in parasitology	63 (13.8)	53 (11.6)	94 (20.6)	100 (21.9)	146 (32.0)
Case studies in immunology and serology	60 (13.2)	55 (12.1)	112 (24.6)	108 (23.7)	121 (26.5)
*Ranking of training needs in research and audit according to pre-determined themes in laboratory specific issues, n = 457*					
Research and audit					
Research proposal development	72 (15.8)	77 (16.9)	102 (22.4)	128 (28.1)	77 (16.9)
Research design	81 (17.8)	75 (16.4)	111 (24.3)	127 (27.9)	62 (13.6)
Research methods	67 (14.7)	72 (15.8)	111 (24.3)	118 (25.9)	88 (19.3)
Grant proposal writing	91 (20.0)	87 (19.1)	99 (21.7)	123 (27.0)	56 (12.3)
Manuscript preparation	101 (22.1)	69 (15.1)	110 (24.1)	121 (26.5)	55 (12.1)

The topics chosen for training in research and audit were summarized in the same manner as the topics in technical competence and were presented as: most important (mean, range 18.1%, 14.7% to 22.1%) (Table 3). Similarly, topics assessed for training under pathophysiology were summarized and presented as: most important (mean, range 14.6%, 11.6% to 17.5%) (Table 3).

### Selected CPD topics according to ranking by participants in relation to area of application in the laboratory with a minimum score of 15%

Table 4 shows topics prioritized for training with a minimum percentage set at 15% which is intentionally selected by the investigators to narrow down the number of selected topics under laboratory issues to few major topics. As in part 3, the topics are classified based on areas of application as technical competence, research skill and pathophysiology knowledge. The total number of specific training needs analyzed within technical competence, research skill and pathophysiology were 68, five and six, respectively. A total of 13 training topics under technical competence, four training topics under research skill and three training topics under pathophysiology were filtered out using the 15% cut-off value criteria applied to the data.

**Table 4:** Priority topics for training according to ranking by participants

Priority topics related to technical competence	%	Priority topics related to research skill	%	Priority topics related to Pathophysiology	%
Perform and interpret DNA/RNA extraction and quantification	33.6	Manuscript preparation	22.1	Case studies in clinical chemistry and molecular diagnostics	20.4
Perform and interpret nucleic acid amplification-based tests/PCR	29.5	Grant proposal writing	20.0	Case studies in clinical microbiology	18.9
Identification of bacterial and mycological species from solid and liquid culture media	23.5	Research design	17.8	Case studies in hematology	16.9
Interpretation of bacterial and mycological colony characteristics	22.4	Research proposal development	15.8		
Perform and interpret antimicrobial susceptibility testing	21.9				
Perform and interpret ELISA testing	20.4				
Perform and interpret hormonal assays	19.1				
Perform and interpret inoculation of microbiological media	19.1				
Perform and interpret culture media preparation	18.0				
Perform and interpret electrolyte analysis	17.8				
Accreditation standards (ENAO, ISO15189)	15.8				
Perform operation and maintenance of clinical chemistry machines	15.4				
Perform collection and examination of other body fluids	15.1				

## Discussion

Continuing professional development is essential in supporting sustained competence of the healthcare workforce [1–5]. This study which aimed at assessing the training needs and educational preferences among medical laboratory professionals in Ethiopia is the first of its kind to be conducted in the country. The study was achieved by utilizing quantitative and qualitative methods aimed at assessing perceived training needs of the training of the participants. All medical laboratory personnel in Ethiopia are mandated to take CPD for licensure purposes as of 2021 [13]. Therefore, the findings in this study would be very valuable in identifying relevant topics for CPD trainings that best meet the needs of the laboratory workforce in the country.

The majority of the respondents in this study picked out training topics in microbiology, research and audit, clinical chemistry and molecular diagnostics in order of importance as the priority areas for training.

However, when specific areas were examined, the three most important topics selected for training in the cross-cutting health issues were health and emerging technology, computer skills and medico-legal issues in order of priority. Laboratory personnel may feel the need to learn more about emerging technology, computer skills and medico-legal issues to successfully cope up with the changing landscape in laboratory practice where technology and computing have become main tools in their professional practice. A recent IFCC article presents in detail the impact of the explosion of new technologies and methods in the future of clinical laboratory practice [14].

Under laboratory specific issues, out of the 11 carefully selected themes for assessing training needs, topics selected in order of importance were: microbiology, research and audit, clinical chemistry and molecular diagnostics, immunology and Serology, pathophysiology, accreditation and quality management, urine and other body fluid analysis, hematology, parasitology, laboratory management, leadership and coaching, immunohematology/blood bank. In a study done by Botswana Health Professions Council over medical laboratory scientists, four top priority areas of training need identified were: (i) quality management systems; (ii) technical competence; (iii) laboratory management, leadership, and coaching; and (iv) pathophysiology, data interpretation, and research [9]. The training needs identified in our survey are slightly different than the study done in Botswana in that most participants in our study prioritized technical competence over accreditation and quality. The reason to this difference could be linked to variation in the time the two studies were conducted. Our study was conducted immediately after the testing gap to COVID-19 posed a major challenge to laboratories across the world and when there is still a heightened sense of technical training need among laboratory professionals.

To further filter out the themes under laboratory specific issues, we regrouped related topics into three categories as technical competence, research skill and pathophysiology knowledge to reassess training priority areas. Moreover, we set a minimum percentage score of 15% for all the training needs evaluated under each category and reevaluated the data. The 15% cut-off score was intentionally selected by the investigators to narrow down the number of selected topics under laboratory issues to few major topics. Based on this, 13 priority training topics were selected under technical competence, four priority topics were selected under research skill and three priority topics were selected under pathophysiology as priority training topics. This analysis revealed that, of the 13 priority training topics identified under technical competence, topics from molecular diagnostics and microbiology topped in the list including training topics such as nucleic acid extraction and detection; and skills related to bacterial and mycological culture.

Manuscript preparation and grant proposal writing was the top two priority training needs identified under topics assessed for skills in research. Similarly, the study participants selected case studies in clinical chemistry and molecular diagnostics, case studies in clinical microbiology and case studies in hematology as priority areas to update their knowledge.

There were some limitations; key among them was that the study did not use a mixed-methods approach (for instance, key informant interviews, and focus groups) to identify CPD training needs which may affect our findings. The use of a variety of methods to confirm the same information by different methods or sources can increase the validity of the findings. However, given that we purposively targeted laboratory personnel at all level of health care delivery and with diverse academic backgrounds, we are confident that the findings we obtained in this study provide rich insights about the training needs of medical laboratory personnel in the country.

## Conclusion

In conclusion, our study identified that CPD programs should focus on topics that address technical competence in microbiology, clinical chemistry and molecular diagnostics. Additionally competencies in research skill and updating knowledge in pathophysiology should also receive due attention in designing trainings. We believe that our finding will allow educators to develop CPD that best meet the needs of the laboratory workforce in the country.

## Supplementary Information

**Additional file 1:** CPD training need assessment tool for Ethiopian Medical Laboratory Science Professionals.

## Data Availability

All materials used in the study are available can be obtained from the corresponding author on reasonable request.

## Abbreviations

AFS: Acid fast staining
ASO: Anti-streptolysin O
CPD: Continuing Professional Development
DNA: Deoxyribonucleic acid
ELISA: Enzyme-linked immunosorbent assay
EMLA: Ethiopian Medical Laboratory Association
ENAO: Ethiopian National Accreditation Organization
EQA: External quality assessment
FMOH: Federal Ministry of Health
IQC: Internal quality control
IRB: Institutional Review Board
LMICs: Low and middle income countries
NGO: Non-governmental organizations
PCR: Polymerase chain reaction
RNA: Ribonucleic acid
SNNPR: Southern Nations, Nationalities and Peoples Region
TNA: Training needs assessment
