# Supplementary material for: Continuing professional development (CPD) training needs assessment for medical laboratory professionals in Ethiopia
Source: Hum Resour Health. 2023 Jun 20;21:47. doi: 10.1186/s12960-023-00837-1 (PMC10283226; doi:10.1186/s12960-023-00837-1)
Supplement: Supplementary file 1 — Additional file 1: CPD training need assessment tool for Ethiopian Medical Laboratory Science Professionals. [file 12960_2023_837_MOESM1_ESM.docx]

# CPD Training Need Assessment Tools for Medical Laboratory Professionals in Ethiopia

**Ethiopian Medical Laboratory Association (EMLA)**

This Assessment tool is designed to collect information on laboratory professionals training need assessment in Ethiopia by EMLA.

**Consent form for study participants**

Dear laboratory professionals, Ethiopian medical laboratory Association (EMLA) in Collaboration with other professional associations and Jhpiego Ethiopia design this baseline assessment on ‘CPD training needs assessment”. CPD has the ambition to maintain competency in professionals and safeguard the public through assurance of competent practice in all health services. FMoH launched the CPD program in all fields of health and linked to licensure of professional practice.

This training need assessment is signed to design a need-based course, to have an overview of the professional’s interests, and organize the CPD implementation at the country level. Considering your day to day practice and your ultimate desire for the laboratory profession, you are requested to rate on the identified area of practice and knowledge.

The data will be collected using an anonymous questionnaire. We anticipate that your participation in this survey will give us your experience and views on this very important subject. The survey will take about 15 to 20 minutes. And the survey process involves no known risks, harm, or threats to you, your family, the institution in which you work, or to the nursing profession. There are no immediate benefits to you for participating in this survey.

Your participation is completely voluntary. You are free to participate or withdraw from this survey at any point in time without any repercussions and to be informed of the survey results. If you agree, please sign this form.

**Do you agree to participate?  Tick on the place provided as**

Yes    o

No    o

**If your answer is yes, go to the next page**

**CPD Training Need Assessment for Laboratory professionals**

**Part I: Demographic data**

| **CPD Training Need Assessment for Laboratory professionals** | | | | |
| --- | --- | --- | --- | --- |
| 1. **General Information** | | | | |
| **S.N** | **Questions** | **Response** | **Remark** |  |
|  | **Region** | **__________** |  |  |
|  | **What is your current work place?** | ☐ Health center  ☐ Primary Hospital  ☐ General hospital  ☐ Specialized hospital  ☐ Health offices  ☐ University/ college  ☐ Non-government  ☐ Private health facility  ☐ Other (specify) _________________ |  |  |
|  | **Which work unit/ward do you work?** | ☐ Hematology  ☐ Immunoheamatology  ☐ Immunology and serology  ☐ Parasitology  ☐ Microbiology  ☐ Urine and body fluid analysis  ☐ Clinical Chemistry and molecular diagnostics  ☐ Other (specify) |  |  |
|  | **Gender:** | ☐ Female ☐ Male |  |  |
|  | **Date of graduation** (/dd/mm/yyyy) |  |  |  |
|  | **Work experience in year** | **____________** |  |  |
|  | **Level of education:** | Diploma in Medical Laboratory Science  BSc in Medical Laboratory Science  MSc in Hematology  MSc in Immunology  MSc in Medical Microbiology  MSc in Medical Parasitology  MSC in Molecular Biology  MSc in Clinical Chemistry  MSc in health laboratory management  PHD  Other (Specify) _______________ |  |  |

**Part II: Cross-cutting issues training need assessment tool**

Considering your day to day practice and experience, how much training you need to maintain competency and how frequent you practice those topics. Rate your level of training need and frequency of activity on scale of 1 to 5 while one indicating no need for training/not ever done and five indicating high level of need for training or routinely done on daily base.

**Required support: -**

**1= Need no support required**: No gap of knowledge or skill: Perform competently

2= Need **minimal of support:** Little gap of knowledge and skill

3= Need a**verage level of support:** Some level of knowledge and skill gap

4= Need **moderate level of support:** significant gap in knowledge and skill

5= Need h**igh level of support:** extensive gap in the knowledge or skill

**Part III: Laboratory specific training need assessment tool**

Considering your day to day practice and experience, how much training you need to maintain competency and how frequent you practice those topics. Rate your level of training need and frequency of activity on scale of 1 to 5 while one indicating no need for training/not ever done and five indicating high level of need for training or routinely done on daily base.

**Required support: -**

**1= Need no support required**: No gap of knowledge or skill: Perform competently

2= Need **minimal of support:** Little gap of knowledge and skill

3= Need a**verage level of support:** Some level of knowledge and skill gap

4= Need **moderate level of support:** significant gap in knowledge and skill

5= Need h**igh level of support:** extensive gap in the knowledge or skill

**Apply skip pattern when it is appropriate**

| **S. No** | **Activities** | **Level of support** | | | | | | | | | | | | | | | **Remark** |
| --- | --- | --- | --- | --- | --- | --- | --- | --- | --- | --- | --- | --- | --- | --- | --- | --- | --- |
|  |  | 1 | 2 | | 3 | | | 4 | | | | | 5 | | | |  |
| **General Issues** | |  |  | |  | | |  | | | | |  | | | |  |
|  | Communication skills in health and Customer care (Interpersonal communication and Provider -client interaction) |  |  | |  | | |  | | | | |  | | | |  |
|  | Medico - legal issues: - (Medico legal system and malpractice issue in the Ethiopian judiciary system, consequences of failure to record medical data) |  |  | |  | | |  | | | | |  | | | |  |
|  | Infection Prevention and Control |  |  | |  | | |  | | | | |  | | | |  |
|  | Basic lifesaving skills and ability to use biohazard safety cabinet |  |  | |  | | |  | | | | |  | | | |  |
|  | Gender in laboratory service delivery |  |  | |  | | |  | | | | |  | | | |  |
|  | Gender based violence – Prevention and response |  |  | |  | | |  | | | | |  | | | |  |
|  | Computer skills (laboratory information system, Microsoft suite) |  |  | |  | | |  | | | | |  | | | |  |
|  | Health and emerging technology  (Technology usage, Tele medicine, online training  HMIS/ DHIS2 and Virtual communication) |  |  | |  | | |  | | | | |  | | | |  |
| **Laboratory management, leadership and coaching** | | | | | | | | | | | | | | | | | |
|  | Ethics and professionalism: - Ethical principles, Professionalism (competencies, dedication, commitment and their application), |  |  | |  | | |  | | | | |  | | | |  |
|  | Critical thinking and decision making |  |  | |  | | |  | | | | |  | | | |  |
|  | Supervision and delegation |  |  | |  | | |  | | | | |  | | | |  |
|  | Data management, report writing and presentation skill |  |  | |  | | |  | | | | |  | | | |  |
|  | Basic cost accounting for clinical laboratory services(example Costing of laboratory tests and procedures, Medical tariffs (billing and coding ) |  |  | |  | | |  | | | | |  | | | |  |
|  | Management of resources and supplies |  |  | |  | | |  | | | | |  | | | |  |
|  | Medical Equipment Management -Proper utilization and preventive maintenance of Medical equipment |  |  | |  | | |  | | | | |  | | | |  |
|  | Work place Stress management and Burn out prevention |  |  | |  | | |  | | | | |  | | | |  |
|  | Team building |  |  | |  | | |  | | | | |  | | | |  |
|  | Strategic planning |  |  | |  | | |  | | | | |  | | | |  |
|  | Rationale selection of testes |  |  | |  | | |  | | | | |  | | | |  |
|  | Training facilitation skill/ Clinical laboratory teaching skills |  |  | |  | | |  | | | | |  | | | |  |
|  | Competency assessment |  |  | |  | | |  | | | | |  | | | |  |
| **Research and audit** | | | | | | | | | | | | | | | | | |
|  | Research proposal development |  |  | |  | | |  | | | | |  | | | |  |
|  | Research design: - Qualitative and quantitative designs |  |  | |  | | |  | | | | |  | | | |  |
|  | Research Methods: - (Data collection methods, tool development and analysis) |  |  | |  | | |  | | | | |  | | | |  |
|  | Grant proposal writing |  |  | |  | | |  | | | | |  | | | |  |
|  | Manuscript preparation |  |  | |  | | |  | | | | |  | | | |  |
| **Technical competence** | | | | | | | | | | | | | | | | | |
| **Accreditation and quality management** | | | | | | | | | | | | | | | | | |
|  | Evaluation and selection of analytical methods and equipment |  |  | |  | | |  | | | | |  | | | |  |
|  | Definition, establishment, and use of reference ranges |  |  | |  | | |  | | | | |  | | | |  |
|  | Point of care testing |  |  | |  | | |  | | | | |  | | | |  |
|  | Method validation and verification |  |  | |  | | |  | | | | |  | | | |  |
|  | Specimen management |  |  | |  | | |  | | | | |  | | | |  |
|  | Accreditation standards (ENAO, ISO15189) |  |  | |  | | |  | | | | |  | | | |  |
|  | Use of internal quality control (IQC) and external quality assessment (EQA) |  |  | |  | | |  | | | | |  | | | |  |
|  | Quality system essentials for medical laboratory |  |  | |  | | |  | | | | |  | | | |  |
|  | Preparation of standard operational procedures |  |  | |  | | |  | | | | |  | | | |  |
|  | Laboratory policies (sample collection policy, sample storage policy…) |  |  | |  | | |  | | | | |  | | | |  |
|  | Techniques to identify and control sources of errors in laboratory procedures |  |  | |  | | |  | | | | |  | | | |  |
|  | Management of non-conformances in laboratory service |  |  | |  | | |  | | | | |  | | | |  |
|  | Able to prepare laboratory reagent and storage(staining solutions, DI and distilled water, chemistry solutions, etc) |  |  | |  | | |  | | | | |  | | | |  |
| **Hematology Laboratory** | | | | | | | | | | | | | | | | | |
|  | Perform and interpret Hemoglobin/Hematocrit Testing |  | |  | | |  | | | |  | | |  | | |  |
|  | Perform and interpret blood cell count (WBC, RBC, PLT…) |  | |  | | |  | | | |  | | |  | | |  |
|  | Perform and interpret WBC differential count |  | |  | | |  | | | |  | | |  | | |  |
|  | Perform and interpret RBC morphology assessment |  | |  | | |  | | | |  | | |  | | |  |
|  | Perform and interpret preparation and examination of thick and thin blood film |  | |  | | |  | | | |  | | |  | | |  |
|  | Perform blood collection (Venus, capillary…) |  | |  | | |  | | | |  | | |  | | |  |
|  | Perform and interpret Coagulation tests (PT, APTT, Fibrinogen…) |  | |  | | |  | | | |  | | |  | | |  |
|  | Perform and interpret flow cytometry procedure |  | |  | | |  | | | |  | | |  | | |  |
| **Immunohematology Laboratory** | | | | | | | | | | | | | | | | | |
|  | Perform and interpret Blood typing and cross matching |  | |  | | |  | | | | |  | | |  | |  |
|  | Perform and interpret preparation of red cell suspension |  | |  | | |  | | | | |  | | |  | |  |
|  | Perform and interpret preparation and storage of blood products |  | |  | | |  | | | | |  | | |  | |  |
|  | Perform and interpret antibody screening and identification |  | |  | | |  | | | | |  | | |  | |  |
| **Immunology and serology laboratory** | | | | | | | | | | | | | | | | | |
|  | Perform and interpret specific and nonspecific treponemal tests (RPR/VDRL, dark field microscopy, FTA-Abs…) |  |  | | |  | | |  | | | |  | | | |  |
|  | Perform and interpret ASO test |  |  | | |  | | |  | | | |  | | | |  |
|  | Perform and interpret widal and weil-felix test |  |  | | |  | | |  | | | |  | | | |  |
|  | Perform and interpret rapid serological tests (HIV, HBV, HCV, malaria…) |  |  | | |  | | |  | | | |  | | | |  |
|  | Perform and interpret pregnancy testing |  |  | | |  | | |  | | | |  | | | |  |
|  | Perform and interpret ELISA testing |  |  | | |  | | |  | | | |  | | | |  |
| **Parasitology laboratory** | | | | | | | | | | | | | | | | | |
|  | Perform and interpret collection and preservation of specimen and slides |  | |  | | |  | | | | |  | | | |  |  |
|  | Perform and interpret direct/wet mount stool examination |  | |  | | |  | | | | |  | | | |  |  |
|  | Perform and interpret stool concentration technique |  | |  | | |  | | | | |  | | | |  |  |
|  | Perform and interpret Examination and Identification of parasites (ova, cyst….) |  | |  | | |  | | | | |  | | | |  |  |
|  | Perform and interpret scotch-tape technique |  | |  | | |  | | | | |  | | | |  |  |
| **Microbiology laboratory** | | | | | | | | | | | | | | | | | |
|  | Perform and interpret gram staining |  | |  | | |  | | | | |  | | | |  |  |
|  | Perform and interpret Acid Fast Staining (AFS) |  | |  | | |  | | | | |  | | | |  |  |
|  | Perform and interpret culture media preparation |  | |  | | |  | | | | |  | | | |  |  |
|  | Perform and interpret inoculation of microbiological media |  | |  | | |  | | | | |  | | | |  |  |
|  | Interpretation of bacterial and mycological colony characteristics |  | |  | | |  | | | | |  | | | |  |  |
|  | Identification of bacterial and mycological species from Solid and Liquid Culture Media |  | |  | | |  | | | | |  | | | |  |  |
|  | Perform and interpret antimicrobial susceptibility testing |  | |  | | |  | | | | |  | | | |  |  |
| **Urine and Body Fluid analysis** | | | | | | | | | | | | | | | | | |
|  | Perform and interpret physical examination of urine |  |  | | |  | | | |  | | |  | | | |  |
|  | Perform and interpret chemical examination of urine |  |  | | |  | | | |  | | |  | | | |  |
|  | Perform and interpret microscopic examination of urine (Cells, casts, crystals…) |  |  | | |  | | | |  | | |  | | | |  |
|  | Perform collection and examination of other body fluids (CSF, Serous, semen…) |  |  | | |  | | | |  | | |  | | | |  |
| **Clinical Chemistry and molecular diagnostics** | | | | | | | | | | | | | | | | | |
|  | Perform operation and maintenance of clinical chemistry machines (spectrophotometer, Immunoassay machines…) |  | |  | | |  | | | | |  | | | |  |  |
|  | Perform and interpret renal function tests (Urea, creatinine…) |  | |  | | |  | | | | |  | | | |  |  |
|  | Perform and interpret liver function test (Albumin, AST, ALT, ALP, Bilirubin) |  | |  | | |  | | | | |  | | | |  |  |
|  | Perform and interpret lipid profile testing (Total cholesterol, TG, HDL-C, LDL-C) |  | |  | | |  | | | | |  | | | |  |  |
|  | Perform and interpret hormonal assays (T3, T4, TSH, testosterone…) |  | |  | | |  | | | | |  | | | |  |  |
|  | Perform and interpret electrolyte analysis (Na+, K+….) |  | |  | | |  | | | | |  | | | |  |  |
|  | Perform and interpret DNA/RNA extraction and quantification |  | |  | | |  | | | | |  | | | |  |  |
|  | Perform and interpret nucleic acid amplification-based tests/PCR (Gene Expert, viral load…) |  | |  | | |  | | | | |  | | | |  |  |
| **Pathophysiology and data interpretation** | | | | | | | | | | | | | | | | | |
|  | Case studies in clinical microbiology |  |  | |  | | |  | | | | |  | | | |  |
|  | Case studies in clinical chemistry & molecular diagnostics |  |  | |  | | |  | | | | |  | | | |  |
|  | Case studies in hematology |  |  | |  | | |  | | | | |  | | | |  |
|  | Case studies in blood banking |  |  | |  | | |  | | | | |  | | | |  |
|  | Case studies in Parasitology |  |  | |  | | |  | | | | |  | | | |  |
|  | Case studies in immunology and serology |  |  | |  | | |  | | | | |  | | | |  |

What CPD topics would you like to access in the future which can be of most benefit for the professional development of your Medical Laboratory practice? (List at least five topics in priority order from the above)

1. ____________________________
2. ____________________________
3. ____________________________
4. ____________________________
5. ____________________________
